# Development of a monoclonal antibody to ITPRIPL1 for immunohistochemical diagnosis of non-small cell lung cancers: accuracy and correlation with CD8^+^ T cell infiltration

**DOI:** 10.3389/fcell.2023.1297211

**Published:** 2023-12-19

**Authors:** Shouyan Deng, Jiawei Shi, Yufan Sun, Yingfei Quan, Zan Shen, Yonggang Wang, Hai Li, Jie Xu

**Affiliations:** ^1^ Division of Gastroenterology and Hepatology, Renji Hospital, School of Medicine, Shanghai Jiao Tong University, Shanghai, China; ^2^ Shanghai Key Laboratory of Medical Epigenetics, International Co-Laboratory of Medical Epigenetics and Metabolism (Ministry of Science and Technology), Institutes of Biomedical Sciences, Fudan University, Shanghai, China; ^3^ BioTroy Therapeutics, Shanghai, China; ^4^ Department of Oncology, Shanghai Sixth People’s Hospital Affiliated to Shanghai Jiao Tong University School of Medicine, Shanghai, China

**Keywords:** cancer biomarker, ITPRIPL1, tumor stage, CD8, T cell infiltration

## Abstract

**Introduction:** Cancer biomarkers are substances or processes highly associated with the presence and progression of cancer, which are applicable for cancer screening, progression surveillance, and prognosis prediction in clinical practice. In our previous studies, we discovered that cancer cells upregulate inositol 1,4,5-triphosphate receptor-interacting protein-like 1 (ITPRIPL1), a natural CD3 ligand, to evade immune surveillance and promote tumor growth. We also developed a monoclonal ITPRIPL1 antibody with high sensitivity and specificity. Here, we explored the application of anti-ITPRIPL1 antibody for auxiliary diagnosis of non-small cell lung cancer (NSCLC).

**Methods:** NSCLC patient tissue samples (*n* = 75) were collected and stained by anti-ITPRIPL1 or anti-CD8 antibodies. After excluding the flaked samples (*n* = 15), we evaluated the expression by intensity (0-3) and extent (0-100%) of staining to generate an h-score for each sample. The expression status was classified into negative (h-score < 20), low-positive (20-99), and high-positive (≥ 100). We compared the h-scores between the solid cancer tissue and stroma and analyzed the correlation between the h-scores of the ITPRIPL1 and CD8 expression *in situ* in adjacent tissue slices.

**Results:** The data suggested ITPRIPL1 is widely overexpressed in NSCLC and positively correlates with tumor stages. We also found that ITPRIPL1 expression is negatively correlated with CD8 staining, which demonstrates that ITPRIPL1 overexpression is indicative of poorer immune infiltration and clinical prognosis. Therefore, we set 50 as the cutoff point of ITPRIPL1 expression H scores to differentiate normal and lung cancer tissues, which is of an excellent sensitivity and specificity score (100% within our sample collection).

**Discussion:** These results highlight the potential of ITPRIPL1 as a proteomic immunohistochemical NSCLC biomarker with possible advantages over the existing NSCLC biomarkers, and the ITPRIPL1 antibody can be applied for accurate diagnosis and prognosis prediction.

## Introduction

In clinical practice, cancer biomarkers, a substance or process indicative of cancer, can be used for cancer epidemiology, diagnosis, progression surveillance, and prognosis prediction (Hung et al., 2022; Chen et al., 2023). Previously discovered cancer biomarkers included genetic, epigenetic, glycomic, proteomic, and imaging (Varghese et al., 2008; Green et al., 2016; Stone, 2017; Black et al., 2019; Dimitrakopoulos et al., 2019; Kang, 2021; Essa et al., 2022; Han et al., 2022; Yao and Zhang, 2023). Cancer biomarkers have been applied in early cancer detection, tumor progression surveillance, and prognosis prediction clinically (Kazmierczak-Siedlecka et al., 2021; Cong et al., 2023; Jiang et al., 2023; LoRusso and Freidlin, 2023). The studies of the cancer biomarker field now focus on developing cost-effectively reliable and robust cancer biomarkers (Cancer immunotherapy: the quest for better biomarkers, 2022).

Inositol 1,4,5-triphosphate receptor-interacting protein-like 1 (ITPRIPL1), a single-transmembrane protein, has been identified as a natural ligand of CD3ε to downregulate T cell function and promote tumor growth in our previous study (submitted). We developed humanized monoclonal anti-ITPRIPL1 antibodies and screened out the antibody with the highest binding affinity and blocking efficacy. The sensitivity and specificity of the antibody were tested by flow cytometry and immunoblot, and the functional activity was tested by *in vivo* studies (submitted).

We found the overexpression of ITPRIPL1 in multiple carcinomas in our previous studies, and in this study, we specifically collected and analyzed NSCLC patient samples with detailed clinical information. We analyzed the expression of ITPRIPL1 by H-score by calculating the intensity (0–3) and extent (0%–100%) of staining to generate an h-score for each sample, defining negative (h-score <20), low-positive (20–99), and high-positive (≥100) (Szczepanski et al., 2023). We found that the ITPRIPL1 expression level was positively correlated with cancer stages and negatively correlated with CD8^+^ T cell infiltration, which indicated poorer clinical outcomes.

To explore the potential of ITPRIPL1 as an auxiliary diagnostic index, we set 50 as the cutoff point of the ITPRIPL1 H-score. We found this value is of 100% sensitivity and specificity according to our samples. In sum, ITPRIPL1 has the potential as a proteomic immunohistochemical cancer biomarker for future clinical concerns, the positive rate of which can suggest the level of CD8 positive T cell infiltration and clinical outcome prognosis.

## Results

### ITPRIPL1 humanized antibody development

**FIGURE 1:**
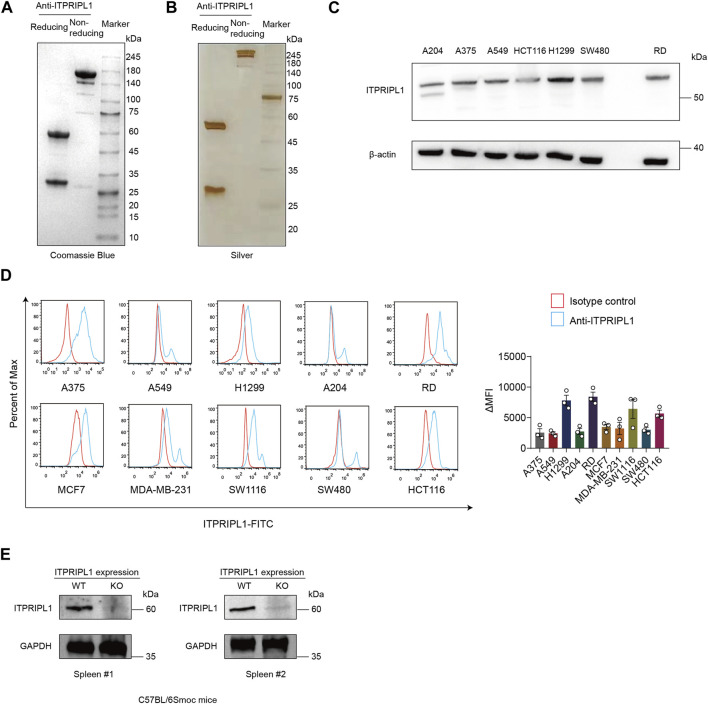
ITPRIPL1 humanized antibody development. **(A)**, Coomassie Blue staining showing the purity of humanized anti-ITPRIPL1 antibody in reducing and non-reducing form (n = 3 independent experiments). **(B)**, Silver staining showing the purity of the humanized anti-ITPRIPL1 antibody in reducing and non-reducing form (n = 3 independent experiments). **(C)**, Immunoblot showing the ITPRIPL1 expression in different tumor cell lines and the specificity of humanized anti-ITPRIPL1 antibody (n = 3 independent experiments). **(D)**, FACS showing the direct binding between humanized anti-ITPRIPL1 antibody and the expression in different tumor cell lines (n = 3 independent experiments). **(E)**, Immunoblot showing lack of bands in the spleen and liver samples extracted from ITPRIPL1 KO mice in comparison with the wild type mice samples (n = 3 independent experiments). Data are mean ± s.e.m.

We developed humanized monoclonal anti-ITPRIPL1 antibodies and selected the antibody with the highest affinity. To demonstrate the purity of our antibody, we performed Coomassie Blue stain and found the purity of the anti-ITPRIPL1 antibody was more than 95% (Figure 1A, Supplementary Figure S1A). The purity of the anti-ITPRIPL1 antibody was further confirmed by silver staining (Figure 1B, Supplementary Figure S1B). To validate the specificity of the anti-ITPRIPL1 antibody further, we performed immunoblot and flow cytometry on different human tumors, including melanoma A375, rhabdomyosarcoma A-204, RD, non-small cell lung cancer A549, H1299, breast cancer MBA-MD-231, MCF7 and colorectal carcinoma HCT116, SW480, SW1116 (Figures 1C, D, Supplementary Figure S1C). The results indicated overexpression of ITPRIPL1 in various human cancer cell lines. The expression level was consistent with the transcriptomic datasets from the Cancer Cell Line Encyclopedia (CCLE) concerning the ITPRIPL1 mRNA levels in different human cancer cell lines and our previous studies. The low background and single protein band also demonstrated excellent binding specificity of the anti-ITPRIPL1 antibody. Furthermore, to exclude the possibility that the band recognized by the antibody might be a different protein, we extracted spleen from ITPRIPL1^−/−^ mice to perform immunoblot on the sample lysates in comparison with the respective organ samples of wild-type C57BL/6Smoc mice (Figure 1E, Supplementary Figure S1D). The lack of bands in the ITPRIPL1 knockout mouse samples also suggested excellent specificity of the developed anti-ITPRIPL1 antibody.

### The overexpression of ITPRIPL1 in most NSCLC patients

**FIGURE 2:**
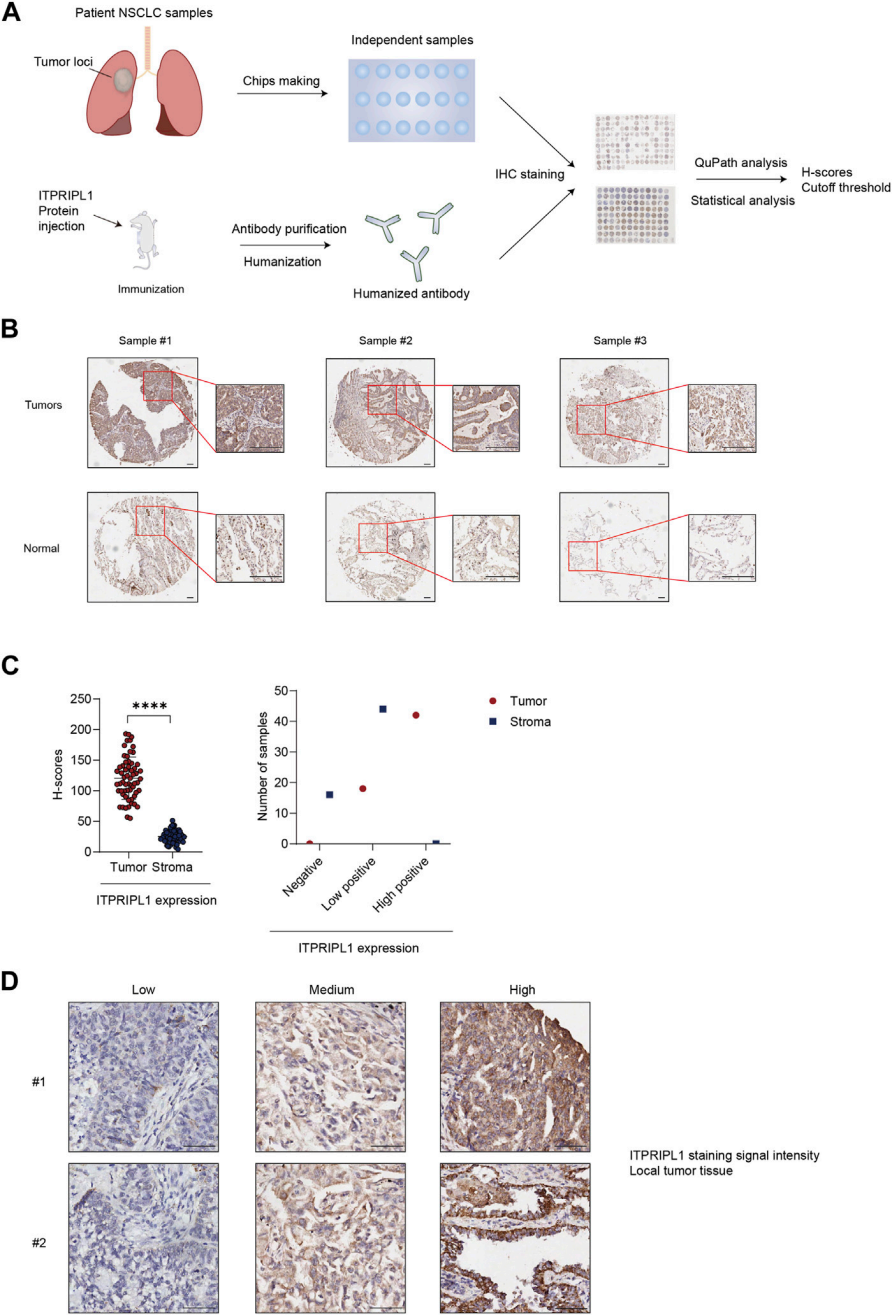
ITPRIPL1 is overexpressed in NSCLC solid tumor tissues in comparison with stroma. **(A)**, Schematic view of the experimental design. **(B)**, Representative images of tumors ITPRIPL1 expression compared with normal tissues (n = 60 groups of samples, 5X and 20X microscopic views, scale bar = 100 μm). **(C)**, ITPRIPL1 H-score calculation and statistical categorization of the solid tumor samples and stroma (n = 60 groups of samples). **(D)**, Representative images of samples showing local tumor tissues stained by anti-ITPRIPL1 with different ITPRIPL1 signal intensities (20X microscopic views, scale bar = 100 μm). *: *p* < 0.05; **: *p* < 0.01; ***: *p* < 0.001; ****: *p* < 0.0001. *p* values are calculated by paired sample *t*-test. Data are mean ± s.e.m.

To evaluate the ITPRIPL1 expression level in different carcinomas, we first collected patient samples of carcinomas of the thyroid gland, esophagus, breast, lung, pancreatic duct, colon, rectum, urinary system, reproductive system, and glioma, each carcinoma type represented by at least 50 samples, and performed immunohistochemistry (IHC) with our humanized antibody (submitted). ITPRIPL1 was widely overexpressed in these patient samples compared to the normal and adjacent tissues. Considering the excellent response to immunotherapy, we focused on non-small cell lung cancer (NSCLC) patients. To precisely evaluate the ITPRIPL1 expression in NSCLC patients, we collected 75 groups of paired patient tissue samples (the tumor tissue and respective paracancerous tissue) and performed immunohistochemistry with our anti-ITPRIPL1 antibody. Of these 60 sample groups in total were analyzed after excluding flaked samples (Figure 2A; Table.1; Supplementary Table S1). We summarized the primary information for the numbers studied in Table.1. The details of the patient information were summarized in Supplementary Table S1. The microscopic views suggested significant ITPRIPL1 upregulation in tumors in comparison to paracancerous tissues (Figure 2B). To quantify the overexpression status of ITPRIPL1, we analyzed the IHC positive scores of the tumors compared with the stroma. The results demonstrated a significant difference in the ITPRIPL1 expressions (Figure 2C). The statistics revealed 0 negative, 18 low-positive, 42 high-positive tumor samples, compared to 16 negative, 44 low-positive, 0 high-positive stroma samples. The difference in the ITPRIPL1 positive rate between solid tumor tissues and stroma suggested notable upregulation of ITPRIPL1 during malignant transformation and a close association between ITPRIPL1 and malignancy. To validate the IHC-staining specificity of the anti-ITPRIPL1 antibody, we analyzed local tumor tissues with different DAB signal intensities (Figure 2D). The representative images showing different DAB signal intensities in different local tumor tissues confirmed the specificity of the anti-ITPRIPL1 antibody. Since the tumor microenvironment consisted of various types of cells, including fibroblasts, immune cells, and blood cells (Park et al., 2023; Su et al., 2023; Zhang et al., 2023), we consulted the ProteinAtlas database (https://www.proteinatlas.org/ENSG00000198885-ITPRIPL1) to consider ITPRIPL1 expression in such host cells contained within the tumor tissues. The database showed minimal ITPRIPL1 expression in normal human tissues, except for the testes. Notably, the primary immune cluster with ITPRIPL1 enrichment within tumor tissues was MAIT T cells, the upregulation of which may promote tumorigenesis by suppressing T cells and NK cells (Berzins et al., 2020; Yan et al., 2020). Our data, combined with results from the ProteinAtlas database indicated the upregulation of ITPRIPL1 signal in NSCLC tumors is largely due to the overexpression of this marker in tumor cells rather than in stromal cells within the tumor microenvironment.

**TABLE 1:** A brief summary of the number of patient samples enrolled in the IHC staining approach.

Tumor classification	Tumor stage	Enrolled samples	Flaked samples	Analyzed samples	Total studied samples
NSCLC	StageIA	13	4	9	9
NSCLC	StageIB	20	4	16	25
NSCLC	StageIIA	11	4	7	32
NSCLC	StageIIB	4	0	4	36
NSCLC	StageIIIA	8	2	6	42
NSCLC	StageIIIB	17	1	16	58
NSCLC	StageIV	2	0	2	60

Thus, ITPRIPL1 appears to overexpress in the majority of NSCLC patient tumors and can be detected by our humanized antibody, representing a hallmark of a proteomic biomarker.

### ITPRIPL1 expression is negatively correlated with T-cell infiltration

**FIGURE 3:**
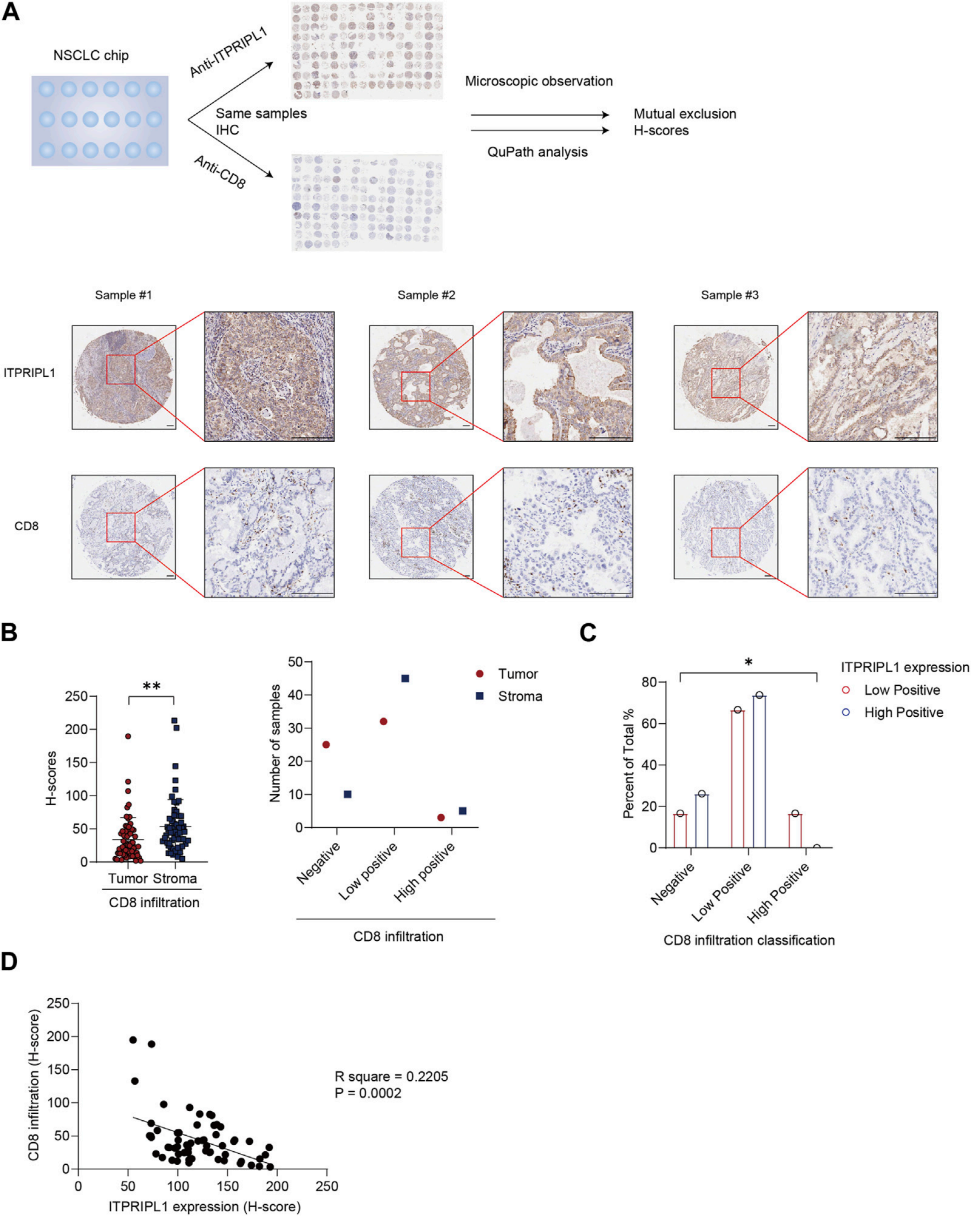
ITPRIPL1 is negatively correlated with CD8 positive T cells infiltration in NSCLCs. **(A)**, Schematic view of the experimental design with representative images of ITPRIPL1 and CD8 in the same tumor samples (n = 60 groups of samples, 5X and 20X microscopic views, scale bar = 100 μm). **(B)**, CD8 H-score calculation and statistical categorization of the solid tumor samples and stroma (n = 60 groups of samples). **(C)**, CD8 infiltration classification among samples with different ITPRIPL1 expression (n = 18 groups of samples for low positive ITPRIPL1 expression and n = 42 groups of samples for high positive ITPRIPL1 expression). **(D)**, The linear correlation between ITPRIPL1 and CD8 in each tumor sample. *: *p* < 0.05; **: *p* < 0.01; ***: *p* < 0.001; ****: *p* < 0.0001. *p* values are calculated by paired sample *t*-test for Figure 3B and one-way ANOVA for Figure 3C. Data are mean ± s.e.m.

In our previous study, we have found ITPRIPL1 can impair T cell function and downregulate anti-tumor immune response (submitted). In this study, we detected a mutually exclusive pattern between ITPRIPL1 expression and CD8^+^ T cell infiltration (Figure 3A). We reanalyzed our data by comparing the expressions of ITPRIPL1 and CD8 in NSCLC patient samples. We performed CD8 IHC staining on adjacent sections of the same tumor samples that we utilized for ITPRIPL1 IHC staining. The microscopic images indicated mutual exclusion between ITPRIPL1 and CD8 in the tumor samples, with substantially decreased CD8 infiltration in high-positive ITPRIPL1 samples in comparison with low-positive ITPRIPL1 samples (Figures 3B, C). Moreover, linear regression analysis revealed a distinct negative correlation between ITPRIPL1 expression and CD8 infiltration (Figure 3D). Immune evasion, associated with a cold tumor microenvironment, was reported as a hallmark of cancer (Hanahan and Weinberg, 2011; Hanahan, 2022). CD8^+^ T cell infiltration was positively correlated with patient survival and clinical prognosis. The negative correlation between ITPRIPL1 and CD8 expression suggests that ITPRIPL1 expression may indicate a “cold” tumor microenvironment as ITPRIPL1 is associated with a low CD8^+^ signal.

### ITPRIPL1 is indicative of tumor progression and prognosis

**FIGURE 4:**
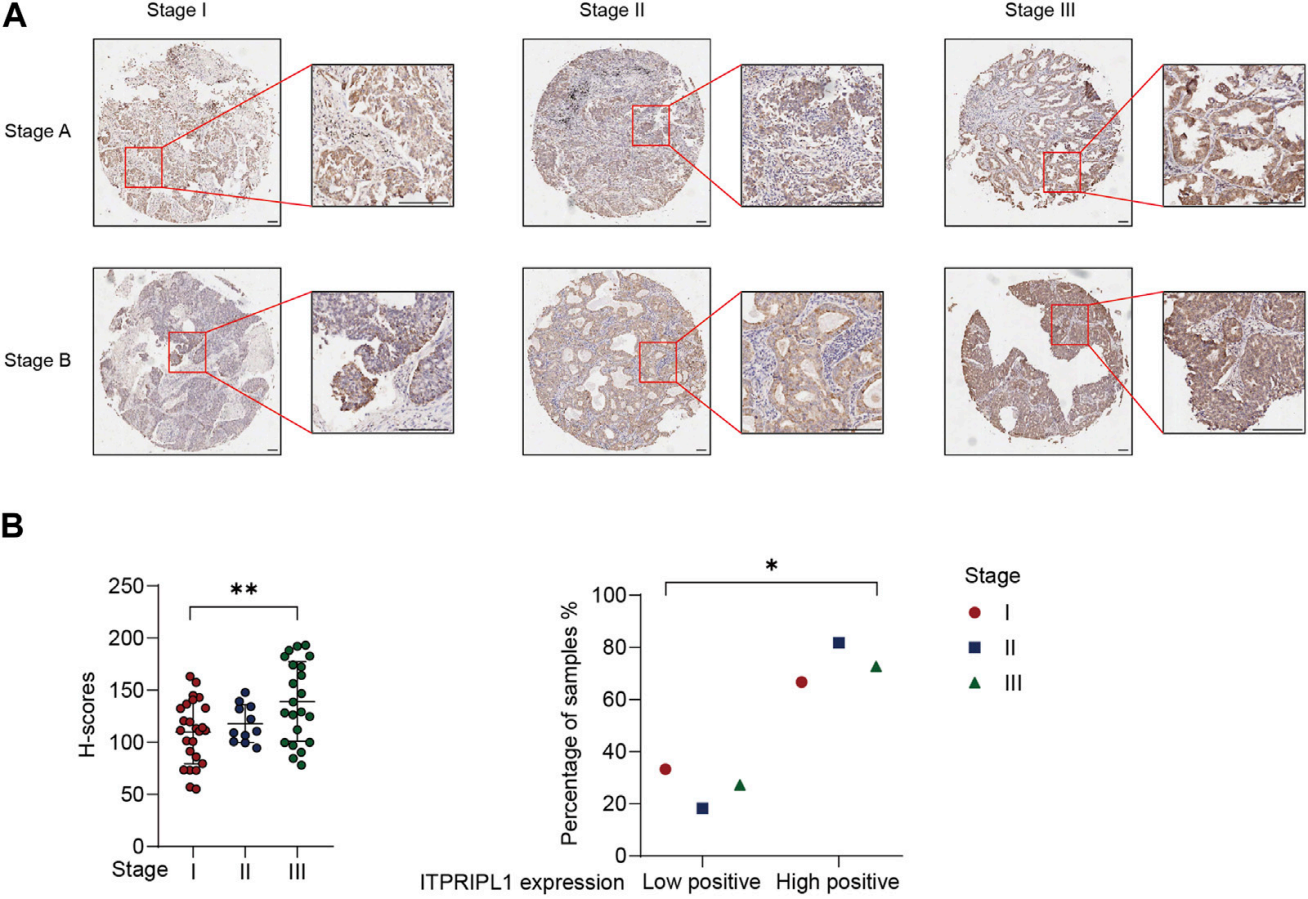
ITPRIPL1 expression is positively correlated with tumor stage. **(A)**, Representative images of ITPRIPL1 expression in different NSCLC stages (n = 25 for stage I, n = 11 for stage II, n = 22 for stage III, 5X and 20X microscopic views, scale bar = 100 μm, Stage A&B stand for the respective tumor stages (e.g., Stage IA for row1 column1, Stage IB for row2 column1 and Stage IIB for row1 Column2)). **(B)**, H-score calculation and statistical categorization of ITPRIPL1 expression in different NSCLC stages (n = 25 for stage I, n = 11 for stage II, n = 22 for stage III). *: *p* < 0.05; **: *p* < 0.01; ***: *p* < 0.001; ****: *p* < 0.0001. *p* values are calculated by one-way ANOVA. Data are mean ± s.e.m.

We demonstrated a negative correlation between ITPRIPL1 and CD8. Poor immune infiltration and the “cold” tumor microenvironment are associated with aggressive tumor progression and poor prognosis (Pickup et al., 2013). To evaluate the correlation between ITPRIPL1 expression and tumor progression, we further collected the detailed stage information of our patients. We compared the ITPRIPL1 expressions in different stages of non-metastatic NSCLC tumors. The ITPRIPL1 expression was positively correlated with tumor stages (Figure 4A). Higher IHC positive scores correlated with higher tumor stages (Figure 4B). Tumor stages are crucial for cancer evaluation and treatment choice. Higher tumor stages are indicative of accelerating tumor progression, greater invasiveness, higher chances of metastasis, and poorer patient prognosis (Filner and Ost, 2008; Brierley et al., 2016; Detterbeck et al., 2022). Therefore, ITPRIPL1 is indicative of more aggressive tumor progression and poorer prognosis.

### ITPRIPL1 is an accurate biomarker for tumor diagnosis

**FIGURE 5:**
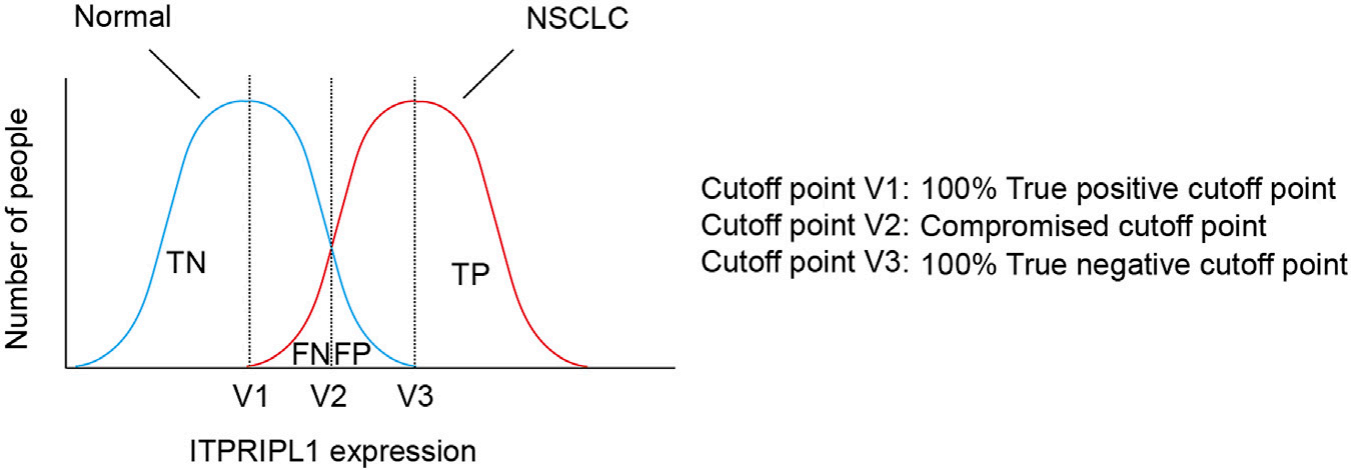
The selection of cutoff point of ITPRIPL1 expression for NSCLC diagnosis. Schematic view for the meanings of different cutoff points.

Considering the positive correlation between ITPRIPL1 expression and tumor stages, we further explored the possibility of setting ITPRIPL1 as an aided diagnostic index. We set the H-score of ITPRIPL1 at the value of 50 as the cutoff point for tumor *versus* normal tissue. Such a value indicates 100% sensitivity and 100% specificity limited to our samples (Figure 5). The accuracy and precision of the ITPRIPL1 positive rate as an immunohistochemical diagnostic biomarker suggests the potential of ITPRIPL1 as an auxiliary method for tumor diagnosis.

## Discussion

The discovery and development of cancer biomarkers have revolutionized the early detection and prognosis prediction of cancers. This study reports that a newly discovered immune checkpoint, ITPRIPL1, can also be leveraged as a proteomic biomarker for tumor diagnosis, prognosis prediction, and relation to CD8^+^ T cell infiltration. This study reports the development of a humanized ITPRIPL1 antibody and its applications. The application of anti-ITPRIPL1 reveals overexpression of ITPRIPL1 in NSCLCs, with correlation with immune infiltration, tumor progression, clinical prognosis, and tumor diagnosis. The database showed upregulated ITPRIPL1 mRNA expression in different cancers, and we confirmed the overexpression of ITPRIPL1 at the protein level, especially in tumors from NSCLC patients. We further identified reduced infiltration with CD8^+^ T cells in NSCLCs, representing the characteristic immunological signature associated with ITPRIPL1 overexpression. The wide overexpression of ITPRIPL1 in cancers made it valuable for assessing tumorigenesis and immune infiltration.

Predicting clinical outcomes is a significant application of cancer biomarkers, and ITPRIPL1 matches the trait. The upregulation of ITPRIPL1 in cancers indicates less sensitivity to the existing immunotherapy, likely related to immune evasion. Previous clinical trials have found that high ITPRIPL1 expression was correlated with worse PD-L1 response [Deng, submitted]. Moreover, the analysis of patients’ clinical information reveals a positive correlation between ITPRIPL1 expression and tumor stages. Clinically higher tumor stages are associated with poorer clinical outcomes and events (Secher et al., 2023). The 5-year survival rate varies significantly in different tumor stages (Li et al., 2022). As for NSCLC, the 5-year survival rates for stage I, II, III, and IV are around 55%, 35%, 15%, and 5% respectively (Di Girolamo et al., 2018). The positive correlation between ITPRIPL1 expression and tumor stages suggests a negative correlation between ITPRIPL1 and prognosis. Collectively, ITPRIPL1 expression can be indicative of clinical outcomes.

Aided Tumor diagnosis is another essential application of biomarkers, and the immunohistochemical positive ITPRIPL1 rate can be applied for improved tumor diagnosis. The excellent accuracy of the ITPRIPL1 H-score for tumor diagnosis made it valuable for testing the ITPRIPL1 level during screening in high-risk patient populations. ITPRIPL1 expression can be easily approached by simply staining pathological samples with ITPRIPL1 humanized antibody. The convenience and cost-effectiveness make ITPRIPL1 a valuable proteomic immunohistochemical biomarker.

ITPRIPL1 may have several advantages over the existing NSCLC biomarkers, such as carcinoembryonic antigen (CEA), carbohydrate antigen (CA) 125, and CYFRA21-1 (Muley et al., 2008; Cedres et al., 2011; Dogan et al., 2014; Chen et al., 2015; Dal Bello et al., 2019). The high immunohistochemical positivity of ITPRIPL1 in NSCLC tumor samples with negative staining in most normal lung tissues suggests potentially better sensitivity and specificity. Additionally, the IHC staining of ITPRIPL1 in tumor tissues is relatively more homogenous, which could assist in delineating the precise boundaries of solid tumors. Furthermore, the immunohistochemical positivity of ITPRIPL1 might be crucial for eligible patients receiving anti-ITPRIPL1 treatment, which is irreplaceable by other NSCLC biomarkers regarding this aspect. However, further research is needed to confirm these potential advantages.

To summarize, ITPRIPL1, our previously discovered immune checkpoint, is also a remarkable NSCLC biomarker. Detecting ITPRIPL1 in tumors can contribute to better diagnosis, CD8 positive T cell infiltration revelation, disease progression surveillance, and prognosis prediction.

## Materials and methods

### Antibodies and reagents

The primary antibody for CD8α (ab245118, abcam), β-actin (ab8226, abcam), GAPDH (ab9485, abcam), and secondary antibodies for Human IgG Fc (A0170, Sigma), Rabbit IgG Fc (31463, Invitrogen) were all commercially available. Reagents involving dimethyl benzene, paraffin wax were provided from Fudan University platform; DAB (DA1010, Solarbio), Citrate sodium (C1032, Solarbio), C57BL/6Smoc-ITPRIPL1em1Smoc mice (Shanghai Model Organisms Center, Inc.), and C57BL/6Smoc mice (SM-001, Shanghai Model Organisms Center, Inc.) were purchased from indicated suppliers.

### Mice organ extraction and protein sample preparation

At the age of 8 weeks, healthy male C57BL/6Smoc-ITPRIPL1em1Smoc or C57BL/6Smoc mice were sacrificed and dissected immediately. The spleen was cut into slices by scissors and put into 1.5 mL EP tubes with two 3 mm grinding magnetic beads. In each tube was added 100 mL strong RIPA lysis buffer (P0013B, Beyotime) with 1% proteinase and phosphatase inhibitors cocktail (ThermoFisher Scientific). The tubes were put into TissueLyser II (QIAGEN) and smashed into tissue homogenate. The homogenate was centrifuged at 12000 rpm for 30 min and the supernatant was mixed with 5X SDS-PAGE loading buffer (Beyotime) to prepare for further experiments.

### Plasmids construction

The sequences of Anti-ITPRIPL1 humanized antibodies were derived from hybridomas and modified according to humanization tools, which were purchased from General Biol, generated by inserting synthesized cDNA into the pcDNA3.1 vector, using the EcoRI/XhoI MCS. All vectors were checked by sequencing and western bolt with specific antibodies in which the observed molecular weights were in concordance with the predicted molecular weights.

### Transfection of plasmids

HEK293 cells were seeded in 6-well plates to reach a density of 50–70% at the time of transfection. 24 h later, transfection was performed using 1.5 μg plasmid together with 4.5 μL FuGENE HD (Promega) and 100 μL Opti-MEM per well according to the manufacturer’s guidance. The negative control in each experiment was cells mock-transfected with an empty control vector.

### Antibody purification

After transfection, the HEK293 cells were collected and rotated at 1000 rpm for 5 min to collect the supernatant only. The supernatant was mixed with protein A beads (Smart-Lifesciences) and slowly rotated at 4°C overnight. The mixture was put into an affinity column. The antibodies were washed down by eluent. The eluates were collected and concentration was determined by Nano-300.

### Antibody purity tests

The antibody purity was directly tested by Coomassie Blue stain. The filtered antibody solution was mixed with 5x reducing or non-reducing loading buffer (Beyotime) and boiled at 100°C for 15 min. The solution was cooled down to room temperature and run in appropriate concentrations of SDS–PAGE. The gel was then stained with an appropriate amount of Coomassie Blue solution under room temperature for 30 min, and discolored by methanol at room temperature overnight. The gel was scanned by ChamGel 5000 (SageCreation).

### Immunoblotting

Cells were lysed with RIPA buffer (Beyotime) supplemented with 1% proteinase and phosphatase inhibitors cocktail (ThermoFisher Scientific). The collected cell lysates were centrifuged for 15 min at 12000rpm (4°C). The supernatant was reserved and the protein concentration was determined with a BCA Protein Assay Kit (ThermoFisher Scientific). 5 × SDS-PAGE loading buffer (Applied Cells, Inc.) was diluted to 1 × with protein sample and heated at 100°C for 8 min. The protein extracts were subjected to appropriate concentrations of SDS–PAGE for electrophoresis and transferred to PVDF membranes (Bio-Rad). Membranes were blocked with 5% bovine serum albumin (ThermoFisher Scientific) for 1 h under room temperature, and then incubated with the primary antibodies overnight at four degrees. Membranes were incubated with secondary HRP-conjugated antibodies (KANGCHEN) at room temperature for 1 h. Before and after the incubation, the membranes were washed five times with TBST and then examined with a Minichemi imaging system (PerkinElmer).

### Coomassie Blue staining

After SDS-PAGE electrophoresis, 1) Put the gels into 50 mL deionized water and heat at high temperature by microwave for 3 min 2) Change the water and repeat step 1.3) Immerse the gels into 20 mL Coomassie Blue Fast Staining Solution (P0017, Beyotime) and rotate at 40 rpm under room temperature for 30 min 4) Add 100 mL deionized water and rotate at 40 rpm under room temperature and change the water every 15 min until the background turns clear.

### Silver staining

All reagents were purchased from the fast silver staining kit, P0017S, Beyotime. After SDS-PAGE electrophoresis, 1) Put the gels into 100 mL fixation liquid and rotate at 60 rpm under room temperature overnight. 2) Wash the gels with 30% ethanol and rotate at 60 rpm under room temperature for 10 min 3) Wash the gels with Milli-Q pure water and rotate at 60 rpm under room temperature for 10 min 4) Add 100 mL silver spiked solution and rotate at 60 rpm under room temperature for 2 min 5) Wash the gel with 200 mL Milli-Q pure water twice, each rotated at 60 rpm under room temperature for 1 min 6) Add 100 mL silver solution and rotate at 60 rpm under room temperature for 10 min 7) Wash the gel with 100 mL Milli-Q pure water twice and rotate at 60 rpm under room temperature for 1 min 8) Add 100 mL silver coloring solution and rotate at 60 rpm under room temperature for 10 min 9) Add 100 mL termination solution and rotate at 60 rpm under room temperature for 10 min 10) Wash the gel with 100 mL Milli-Q pure water and rotate at 60 rpm under room temperature for 5 min.

### Flow cytometry

The cells (1 × 10^6^/ml) were added 100 μL per well a flat-bottom 96-well plate (Costar) with 10 μg/mL anti-ITPRIPL1 antibody. We incubated the plate at 37°C under 5% CO2 for 30 min. After incubation, we washed the samples with flow cytometry staining buffer (Invitrogen) three times. Then we diluted the anti-human IgG Fc region Alexa Flour 488 (Invitrogen) fluorescent antibody at suggested concentrations according to the supplier in staining buffer and added 200 μL to each sample. We incubated them on ice for 30 min, protected from light. Next, after being washed with staining buffer three times, we transferred the samples into single tubes (Falcon) and analyzed them by NeonSYS (Beamcyte). We applied FlowJo V10 to analyze the data.

### Immunohistochemistry

The tumor and normal tissue specimens were extracted from patients and immediately stored in a formaldehyde solution (Solarbio). The samples were embedded with paraffin wax and cut into slides. The samples were dewaxed by sequentially being immersed into dimethyl benzene, 100% ethanol, 90% ethanol, 70% ethanol, 50% ethanol, pure water, and PBS, twice for 5 min. The samples were mixed with 3% H_2_O_2_ for 15 min to inactivate the endogenous peroxidase enzyme. The samples were immersed into citrate sodium and put into a microwave oven, sequentially undergoing high heat for 2 min, and medium heat for 10 min. The samples were then cooled down to room temperature and incubated with antibodies against ITPRIPL1 and CD8α at 4°C overnight. After three PBS washes, the tissues were then incubated with a biotin-conjugated secondary antibody, followed by avidin–biotin–peroxidase complex. Visualization was performed using aminoethyl carbazole chromogen.

### Digital image analysis

IHC images of ITPRIPL1 and CD8 were visualized at × 20 magnification using Aperio ImageScope v12.1.0.5029 and iViewer v7.2.7.6alpha. The images were analyzed by Qupath v0.4.3 and the traditional H-score was calculated based on the cell-membrane localized biomarker signal of each tissue sample.

### H-score calculation

IHC images were analyzed by QuPath (v0.4.3), an open-source software for whole-slide analysis. Cells were detected and scored as high, medium, and low based on the average cell DAB signal intensity by a custom script written in the Groovy programming language, which first deconvolves the image into hematoxylin and DAB channels, and then analyze - > cell analysis - > cell detection was used to discriminate individual cells and the respective subcellular compartments. A minimum filter was applied to subtract the background, and cells devoid of a nucleus were excluded and the remaining cells were classified into low, medium, and high DAB signal intensity based on the mean intensity. The script outputs the total detected cells with low, medium, and high DAB signal intensity and calculated H-scores by calculating 1*low% + 2*medium% + 3*high%.

## Data Availability

The original contributions presented in the study are included in the article/Supplementary Material, further inquiries can be directed to the corresponding author.
