# Supplementary figures and images for: Development of a monoclonal antibody to ITPRIPL1 for immunohistochemical diagnosis of non-small cell lung cancers: accuracy and correlation with CD8+ T cell infiltration
Source: Front Cell Dev Biol. 2023 Dec 19;11:1297211. doi: 10.3389/fcell.2023.1297211 (PMC10770237; doi:10.3389/fcell.2023.1297211)

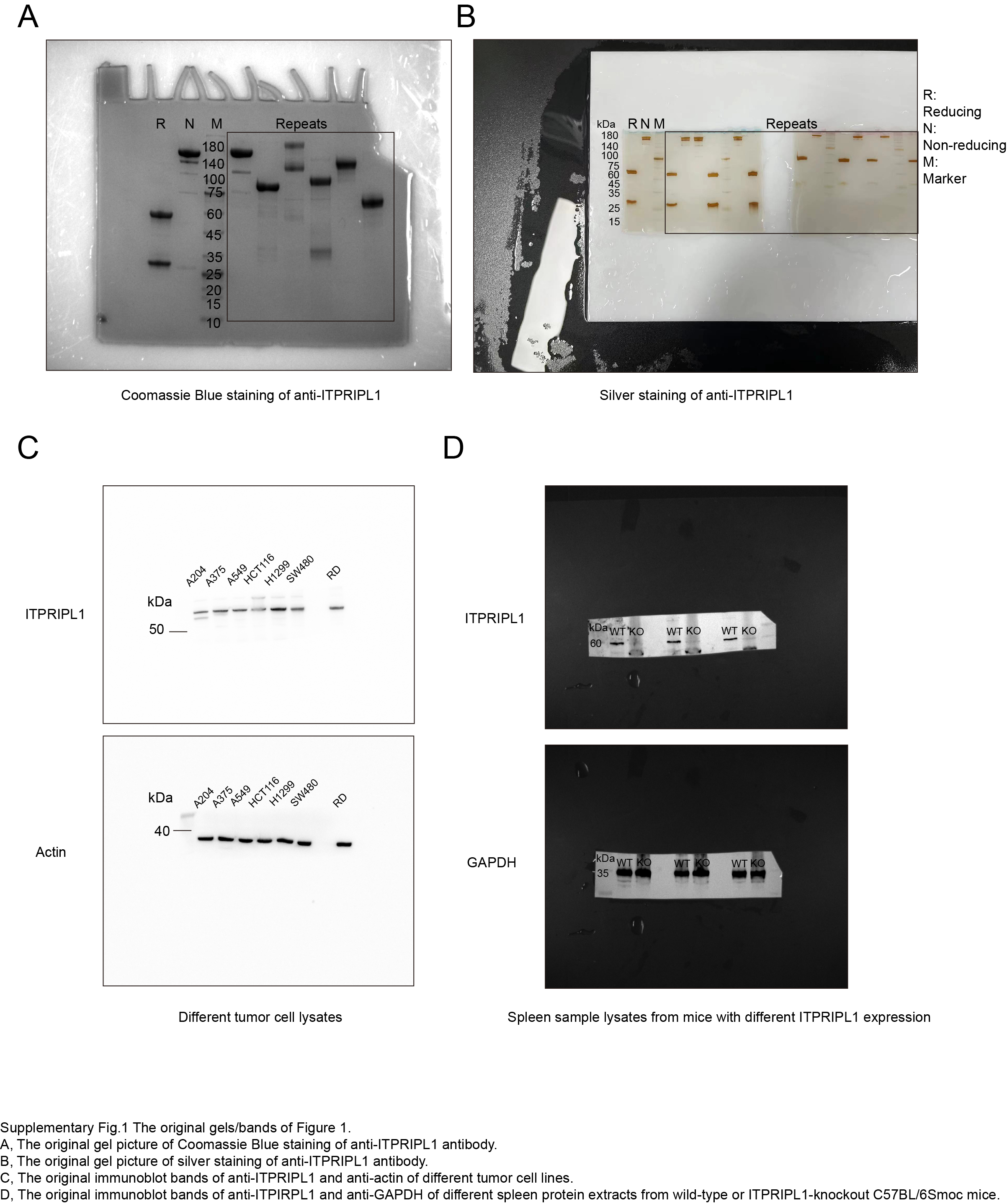

Supplement: Supplementary file 2 [file Image1.JPEG]
